# Concentration of Immunoglobulins in Microfiltration Permeates of Skim Milk: Impact of Transmembrane Pressure and Temperature on the IgG Transmission Using Different Ceramic Membrane Types and Pore Sizes

**DOI:** 10.3390/foods7070101

**Published:** 2018-06-28

**Authors:** Hans-Jürgen Heidebrecht, José Toro-Sierra, Ulrich Kulozik

**Affiliations:** 1 Chair for Food and Bioprocess Engineering, Technical University of Munich, Weihenstephaner Berg 1, 85354 Freising, Germany; toro.jose@live.com (J.T.-S.); ulrich.kulozik@tum.de (U.K.); 2 Kraft Foods R&D Inc./Mondelēz International GmbH, 82008 Unterhaching, Germany; 3 ZIEL Institute for Food & Health, Technical University of Munich, Weihenstephaner Berg 1, 85354 Freising, Germany

**Keywords:** immunoglobulin G, microfiltration, milk protein fractionation, ceramic membranes

## Abstract

The use of bioactive bovine milk immunoglobulins (Ig) has been found to be an alternative treatment for certain human gastrointestinal diseases. Some methodologies have been developed with bovine colostrum. These are considered in laboratory scale and are bound to high cost and limited availability of the raw material. The main challenge remains in obtaining high amounts of active IgG from an available source as mature cow milk by the means of industrial processes. Microfiltration (MF) was chosen as a process variant, which enables a gentle and effective concentration of the Ig fractions (ca. 0.06% in raw milk) while reducing casein and lactose at the same time. Different microfiltration membranes (ceramic standard and gradient), pore sizes (0.14–0.8 µm), transmembrane pressures (0.5–2.5 bar), and temperatures (10, 50 °C) were investigated. The transmission of immunoglobulin G (IgG) and casein during the filtration of raw skim milk (<0.1% fat) was evaluated during batch filtration using a single channel pilot plant. The transmission levels of IgG (~160 kDa) were measured to be at the same level as the reference major whey protein β-Lg (~18 kDa) at all evaluated pore sizes and process parameters despite the large difference in molecular mass of both fractions. Ceramic gradient membranes with a pore sizes of 0.14 µm showed IgG-transmission rates between 45% to 65% while reducing the casein fraction below 1% in the permeates. Contrary to the expectations, a lower pore size of 0.14 µm yielded fluxes up to 35% higher than 0.2 µm MF membranes. It was found that low transmembrane pressures benefit the Ig transmission. Upscaling the presented results to a continuous MF membrane process offers new possibilities for the production of immunoglobulin enriched supplements with well-known processing equipment for large scale milk protein fractionation.

## 1. Introduction

The natural function of the major bovine immunoglobulin G (IgG) class is to agglutinate pathogens and enhance the complement system. It, therefore, protects the calf from pathogens [1]. This function renders immunoglobulins (Ig) an interesting material for various food supplements and pharmaceutical applications. Such products are used to support the immune system, respectively, which may be used to treat gastrointestinal human diseases after immunizing the cow with the relevant antigen [1,2]. Typically, these products are obtained from colostrum and milk, which is secreted by the cow directly a few hours after parturition. Colostrum naturally contains very high immunoglobulin concentrations (2–20%) [3]. However, the downside of using colostrum is that it is only available a few hours after the birth of a calf and it is needed for its breeding, which makes it difficult to collect large quantities. Contrary to colostrum, mature milk is abundant but lower in the immunoglobulin content. For this reason, some researchers used mature cow milk as a starting material to obtain a rennet whey concentrate containing specific Ig for the treatment of *Clostridium difficile* induced diarrhea [4,5,6]. 

However, due to the low concentration of 0.06% to 0.08% (*w*/*w*) of Ig in raw milk, it is necessary to enrich the immunoglobulin fraction to achieve a high enough concentration for certain biological functionalities in humans. In order to do so, one of the first steps is casein removal. While acid casein precipitation might lead to the degradation of the immunoglobulins [7], precipitation by chymosin requires a downstream heating step to inactivate chymosin, which affects the stability of the immunoglobulins [8]. In parallel, rennet whey contains caseinomacropeptide derived from the cleavage of kappa-casein by chymosin, which results in a lower purity of Ig in relation to the total protein content. In contrast, microfiltration (MF) is a comparatively gentle process. The use of nominal pore sizes in the range of 0.1–0.2 µm not only enables the removal of casein micelles but also the remaining fat globules (0.2–6 µm), microorganisms (0.2–15 µm), and somatic cells (6–15 µm). The resulting casein free milk serum, in manufacturing environments referred to as ‘ideal whey’ or ‘native whey’, has the native milk pH and is free from caseinomacropeptide and cheese curd particles. Therefore, it is clear in appearance and, most importantly, virtually free from microorganisms [9,10]. The focus of recent research regarding milk protein fractionation by microfiltration concentrated on the separation of casein micelles and the major whey proteins α-lactalbumin (α-La) and β-lactoglobulin (β-Lg) and/or the quantification of the obtained casein/whey protein ratio in the MF concentrate [11,12,13,14,15,16,17,18,19,20,21]. However, there is a lack of information regarding the performance of the membranes for separating minor whey proteins such as the immunoglobulin fraction. The decisive question originates from the fact that the major immunoglobulin class IgG (146–163 kDa) [3] is approximately 10 times larger in molecular mass when compared to the major whey proteins (14–18 kDa). As far as reported for α-La and β-Lg, a transmission of ca 50% of the available protein in skim milk into the MF permeate can be expected [15]. Two studies showed that it is possible to separate casein and IgG with microfiltration (at a pore size of 0.1 µm) from colostrum, but they reported low recovery rates of 64% [22], respectively. This includes a maximum transmission of 25% for IgG through the membrane [10]. The same group also reported transmission levels above 99% for IgG during the concentration of skim milk with microfiltration [10], which is diametrically different and in disagreement with 30% transmission observed by Le Berre and Daufin (1998) during the filtration of heat-treated and mineral modified milk [23]. 

The aim of this work was to determine whether it is possible to use microfiltration for the fractionation of casein micelles and IgG at high transmission levels. This paper aims to compare, in a quantitative form, the transport of IgG through the MF membrane with the much smaller major whey proteins α-La and β-Lg. In this context, the impact of transmembrane pressure and temperature on the IgG transmission was studied using different membrane types and pore sizes. It was found that ceramic gradient membranes with a pore size of 0.14 µm featured IgG-transmission rates between 45% to 65% while reducing the casein fraction below 1% in the permeates. This is comparable to the transmission of α-La and β-Lg despite the large (up to 10×) differences in molecular mass. The presented results show that upscaling the process to a continuous MF membrane process might offer new possibilities for the production of immunoglobulin-enriched supplements.

## 2. Materials and Methods 

### 2.1. Microfiltration of Skim Milk

Raw skim milk was obtained from local dairy or raw milk directly from a local experimental farm to avoid thermally-induced interactions between casein micelles and immunoglobulins as well as thermal degradation of the immunoglobulins due to pasteurization. The raw milk was separated with a pilot cream separator type MM 1254 D (GEA Westfalia Group GmbH, Oelde, Germany) at 8.000 g and 50 °C to obtain a fat content below 0.1% in the skim milk. The skim milk was either cooled to 4 °C with a plate heat exchanger and processed the following day or was subjected directly to the same microfiltration unit as described by Kühnl et al. [17]. The microfiltration was operated in a crossflow mode with a closed loop using ceramic ISOFLUX^®^ and standard (TAMI Industries, Nyons, France) membranes (support and selective layer titanium dioxide) both with nominal cutoffs of 0.14 µm, 0.2 µm, 0.45 µm, or 0.8 µm. The membrane featured an area of 0.35 m^2^, 1178 mm length, 25 mm diameter, 23 channels, and 3.5 mm equivalent hydraulic diameter per channel. 

### 2.2. Operating Conditions during Microfiltration

Prior to filtration, the membrane was cleaned and conditioned with 0.5% Ultrasil 14 caustic solution (Ecolab, Düsseldorf, Germany) at 60 °C for 20 min. The preheated membrane was extensively flushed with softened water and cooled down to a filtration temperature of 50 °C ± 1 °C or 10 °C ± 1 °C The filtrations were performed in batch mode for 90 min with 30 L of skim milk of which the first 5 L of retentate were discarded to avoid a dilution by rinsing water. Sampling of retentate and permeate from the feed tank was performed, respectively. The permeate pipeline was performed after 0 min, 10 min, 20 min, 30 min, 45 min, 60 min, and 90 min. Samples were analyzed for β-Lg, α-La, BSA and IgG, and casein, as described in Section 2.3. At the same time points, the permeate flux (J) was measured gravimetrically and converted to liters by dividing the values by the density at the equivalent temperature. The MF-process was operated at a transmembrane pressure Δp_TM_ = 1 bar. If not stated otherwise, wall shear stress τ_w_ = 150 Pa ± 5 Pa (equivalent to 2 bar pressure drop). The Δp_™_ was calculated according to Equation (1) with p_inlet_ and p_outlet_ as the pressures at the membrane inlet and outlet, respectively. The pressure on the permeate side p_permeate_ was adjusted with a manual valve to achieve low Δp_TM_.

(1) $$\;P=\frac{p_{\mathrm{inlet}}+p_{\mathrm{outlet}}\;}{2}-p_{\mathrm{permeate}}$$

The transmission (P) was calculated, according to Equation (2), as the ratio of the concentration of the equivalent component in the permeate (C_per_) and retentate (C_ret_).

(2) $$\;P=\frac{C_{\mathrm{perm}}}{C_{\mathrm{ret}}}\;\cdot 100\%$$

After an experiment, the plant was flushed with water and cleaned in a three-step procedure (alkaline-acid-alkaline), which was described by Kühnl et al. [17]. The cleaning efficacy was validated by measuring the water flux at fixed conditions. 

### 2.3. Measurement of Immunoglobulin G with Reversed-Phase High-Performance Liquid Chromatography

The quantitative determination of native IgG, β-Lg, α-La, and blood serum albumin (BSA) in one run was conducted by reversed-phase high-performance liquid chromatography (RP-HPLC) with an elution profile of acetonitrile and water in the mobile phase, according to Kessler and Beyer (1991) [24] with the gradient and column modifications described by Toro-Sierra et al. (2013) [25]. Calibration was carried out using purified bovine IgG, β-Lg, α-La, and blood serum albumin (BSA) (Sigma, Steinheim, Germany). Before analysis, the pH of all samples was adjusted to 4.6 with 1 M HCl in order to precipitate casein micelles and denatured whey proteins. The supernatant of all samples was filtered through 0.45 µm syringe filters and injected into the RP-HPLC.

### 2.4. Measurement of Casein with RP-HPLC

The quantitative determination of the single casein fractions αS1, αS2, β, und κ-Casein was done with RP-HPLC, according to Bonfattti et al. [26] using the buffer according to Bonizzi et al. (2009) [27]. Shortly, 400 μl of sample were mixed with 1600 µL buffer (Tris(hydroxymethyl)aminomethane (TRIS, 0.165 mol L^−1^), urea (8.000 mol L^−1^), sodium-citrate (0.044 mol L^−1^), and β-mercaptoethanol (0.3% v/w). The solution was filtered (0.45 µm) and injected into vials. The separation was carried out with an Agilent Zorbax 300SB-C18 150 × 4.6 mm resin (Agilent Technologies, Böblingen, Germany) and the gradient elution (Eluent A, 10% acetonitrile (1 mL L^−1^ trifluoroacetic acid). Eluent B, 90% acetonitrile (0.7 mL L^−1^ trifluoroacetic) was carried out at 1.2 mL min^−1^.

## 3. Results and Discussion 

### 3.1. IgG Transmission as a Function of Time and Pore Size

**Figure 1:**
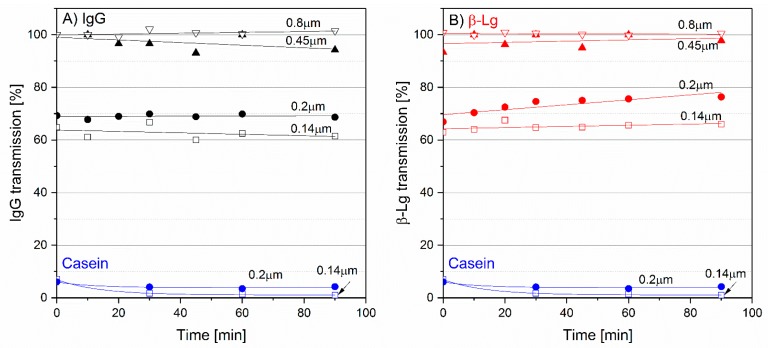
IgG (black) and casein(blue) (**A**) and β-Lg (red) and casein (blue) (**B**) transmission as a function of time at a pore size of 0.14 µm (□) belonging illustrated by the arrow), 0.2 µm (●), 0.45 µm (▲), 0.8 µm (Δ) (gradient membrane, 50 °C). For casein transmission of 0.45 µm and 0.8 µm membranes, see the Supplementary Material.

**Figure 2:**
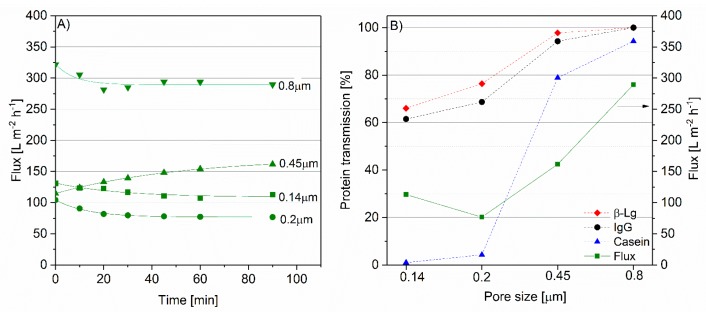
Flux (green) as a function of the time at different pore sizes (**A**), IgG, β-Lg, casein transmission plus flux as a function of pore size at steady state conditions after 90 min of filtration (**B**) (gradient membrane, 50 °C). Arrow indicates the belonging of the flux curve to the right y-axis.

The aim of the microfiltration when used for casein fractionation is to completely retain the casein micelles with a spread in diameter between 50–400 nm (d_50,3_ = 180–200 nm) while maximizing the transmission of the whey proteins ranging from 14 kDa for α-La up to IgG (146–163 kDa) or more for other immunoglobulin fractions. In this way, the ratio of casein to whey proteins is modified from 80:20 (*w*/*w*) as present in raw bovine milk towards higher casein concentrations e.g., 90:10 or 95:5 in the retentate. In milk protein fractionation by microfiltration, the retained proteins form a deposit layer, which influences the filtration performance in terms of flux and protein transmission [11,16]. Therefore, an accurate prediction of the Ig transmission by comparing the nominal pore size of the MF membrane and the molecular size is not possible. Figure 1 shows the IgG (A) and β-Lg (B) as well as casein transmission as a function of time. The flux is presented in Figure 2A,B, which summarizes the data as a function of the different pore sizes at steady state conditions.

**Scheme 1:**
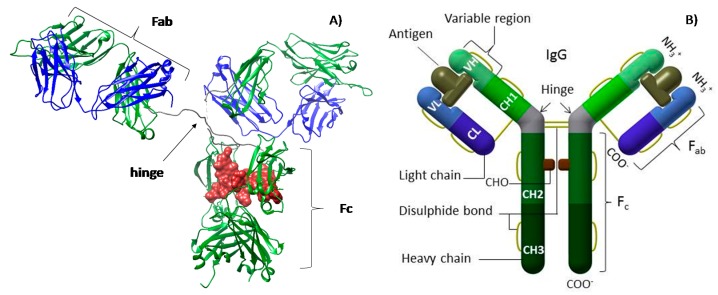
Three dimensional (**A**) and schematic (**B**) structure of IgG. Heavy chains (green), light chains (blue), disulphide bonds (yellow), constant (CH1), and variable region (VH) of the heavy chain, the constant (CL) and variable region (VL) of the light chain, fragment antigen binding (Fab), and the fragment crystallizable (Fc). Three-dimensional structure generated with UCSF Chimera [30] using pdb code 1HZH from the RCSB Protein Data Bank [31]. Schematic structure drawn with AutoCAD and modified from References [28,29].

The transmission levels of IgG (Figure 1A) were found to be at the same level as the reference major whey protein β-Lg (18 kDa) (Figure 1B) at all evaluated pore sizes despite the large difference in the molecular size of both fractions. A reason for the comparatively good transmission behavior of IgG might be its flexibility and shape. IgG consists of two heavy chains and two light chains, which are connected via disulphide bonds (Scheme 1). The constant region (CH1) and variable region (VH) of the heavy chain form together with the constant (CL) and variable region (VL) of the light chain in the fragment antigen-binding (Fab). The constant regions CH2 and CH3 of the two heavy chains form the fragment crystallizable (Fc) [3,28,29]. The Fab and Fc region are connected by a hinge region with no secondary structure (Scheme 1A). From a functional perspective, this gives the molecule a high flexibility so that the two Fab regions can interact with different epitopes of antigens. From a filtration perspective, this flexibility might contribute to the fact that the proteins can pass the deposit layer and membrane-like globular proteins, which is 10 times lower in molecular sizes such as β-Lg.

There was an initial flux decline for membranes with nominal pore sizes of 0.14 µm, 0.2 µm, and 0.8 µm (Figure 2A), which can be attributed to initial fouling during the transition from water to milk [32]. After approximately 30 min, the steady state was reached. Strikingly, the flux increased by ca. 30% when changing to the membrane with the pore size of 0.2 µm to 0.14 µm. This lower flux of the 0.2 µm membrane might be attributed to pore blocking. The casein micelles feature a particle size distribution range of 50–400 nm with a mean of 180–200 nm. This means the average particle size of the casein micelles matches the nominal pore size of the 0.2 µm membrane, which might lead to the blocking of the membrane pores rather than forming a deposit layer on top of the membrane, which can be assumed for the 0.14 µm membrane [33]. Initial pore blocking might also be responsible for the initial low flux for the 0.45 µm membrane, which increased by 30% along the filtration time. 

The ceramic gradient membranes with pore sizes of 0.14 µm and 0.2 µm showed IgG-transmission rates over 60% while reducing the casein fraction below 1% (0.14 µm) and 4 % (0.2 µm) in the permeates, respectively (Figure 2B). For the casein fraction, similar amounts of 1.4% were reported using a pore size of 0.2 µm in the uniform transmembrane pressure mode (UTP) while, at a pore size of 0.1 µm, the casein transmission was negligible [21]. Even though 4% does not seem to be much, due to the different ratio of casein micelles of (80%) to whey proteins (20%) in skim milk, already minor protein transmission led to relatively large impurities in the MF-permeate. Even though typical pore sizes of ceramic membranes for casein whey protein fractionation are between 0.05 and 0.2 µm [34], larger pore sizes were tested because the IgG transmission was expected to be at a lower level at the smaller pore sizes. However, even though the IgG transmission was above 90% for the pore sizes of 0.45 µm and 0.8 µm, the high casein transmission of >75% renders these membranes unsuitable for this fractionation task.

### 3.2. Impact of Membrane Type on IgG Transmission and Flux

**Scheme 2:**
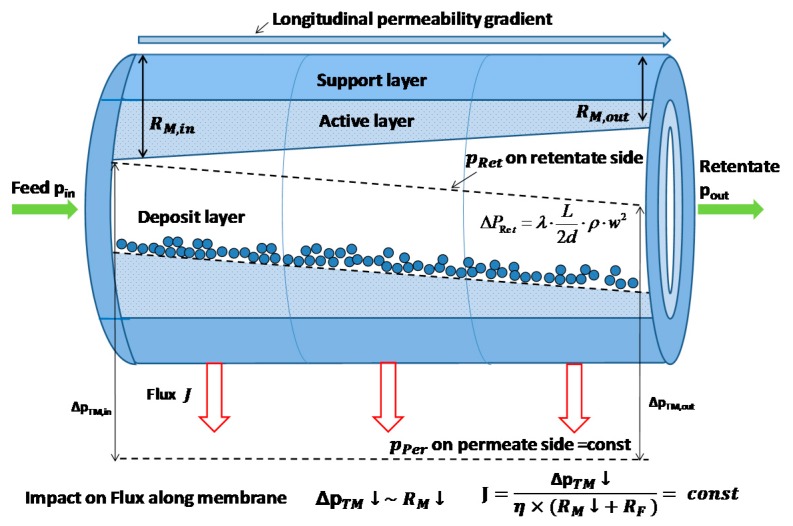
Schematic description of the used gradient membrane. Concept of TAMI Industries, modified from [35]. Flux J, transmembrane pressure Δp_TM_, dynamic viscosity η, membrane resistance R_M_, and fouling resistance R_F_. The indices in and out refer to the membrane inlet and membrane outlet.

**Scheme 3:**
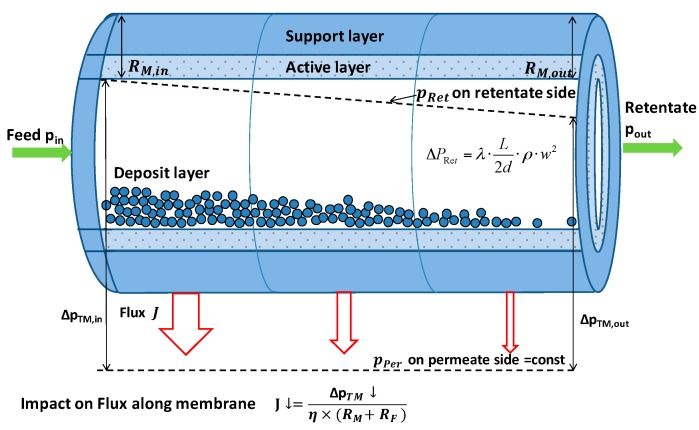
Schematic description of a standard membrane. Flux J, transmembrane pressure Δp_TM_, dynamic viscosity η, membrane resistance R_M_, and fouling resistance R_F_. The indices in and out refer to the membrane inlet and membrane outlet.

The used gradient membranes (Figure 1 and Figure 2) possess a thickness gradient of the selective layer along the membrane, which creates a longitudinal decreasing the membrane resistance (Scheme 2). This means that the higher Δp_TM_ at the membrane inlet compared to the outlet is compensated by a higher membrane resistance at the inlet and a lower resistance at the outlet, which means the permeate volumetric flow is constant along the entire membrane element. By decreasing the membrane resistance corresponding to the decreasing Δp_TM_ along the membrane, the same flux (isoflux) is generated with the objective to improve the overall filtration performance. However, the standard membrane (Scheme 3) does not have a resistance gradient. Therefore, there is a flux decline along the membrane. Moreover, the higher Δp_TM_ at the inlet provokes a more intense deposit layer formation at the inlet, which effects both the flux and protein transmission [16]. However, the retentate pressure drop along the membrane depends on the material, the length of the membrane, the hydrodynamic diameter, the density of the fluid, and the fluid viscosity. As for a given temperature, the density and the membrane characteristic are constant. The pressure drop is directly proportional to the flow velocity. Therefore, the fixed membrane resistance gradient requires a defined pressure drop or crossflow velocity, respectively. Therefore, it renders the operation of the filtration plant inflexible.

**Figure 3:**
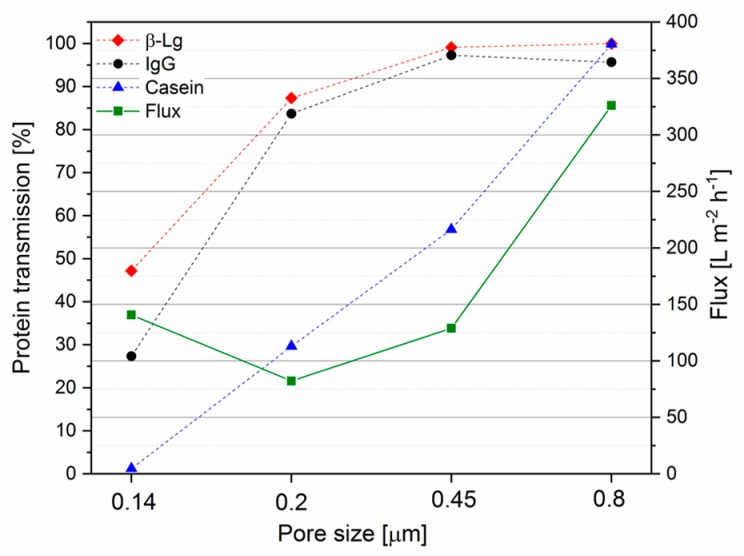
IgG and casein transmission as well as flux function at different pore sizes using ceramic standard membranes at a steady state flux after 90 min of filtration at 50 °C. For time dependent data, see Supplementary Data.

Figure 3 shows the protein transmission and flux as a function of the pore size at steady state conditions using standard membranes. The transmission of IgG was 28% for the 0.14 µm pore size compared to 60% by using the equivalent gradient membrane at the same process conditions. The reason for the inferior performance could be an inhomogeneous deposit (as indicated in Scheme 3). Even though the initial overall permeate fluxes with both membranes were comparable (138 L m^−2^ h^−1^ versus 131 L m^−2^ h^−1^, see Supplementary Material Figure S2), during the transition from water to milk, the flux at the membrane inlet should be higher for the standard membrane due to the missing additional membrane resistance R_m_. Therefore, the convective transport of particles to the membrane surface is higher, which in turn leads to a more intense deposit layer formation especially at the inlet of the membrane. This negatively effects the overall protein transmission. The flux was similar for both membranes. For the other pore sizes, the casein transmission was too high in order to allow the fractionation of IgG and casein. In conclusion, the separation of IgG and casein with a pore size of 0.14 µm using a conventional membrane is feasible. Nevertheless, due to their better performance, the gradient membranes were used for further experiments.

### 3.3. Impact of Transmembrane Pressure on IgG Transmissionand Flux at Different Pore Sizes

**Figure 4:**
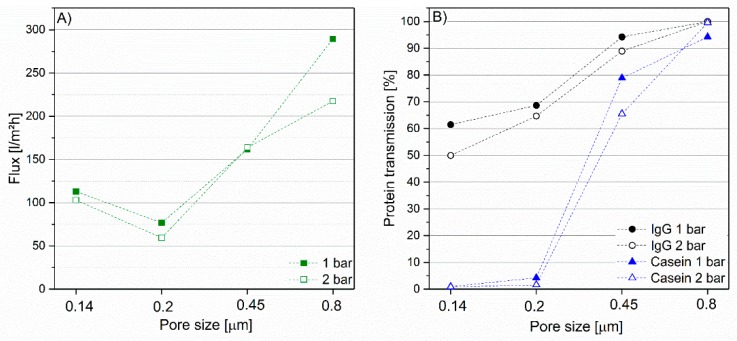
Flux (**A**) and IgG plus casein transmission (**B**) as a function of pore size at 1 bar (filled symbols) and 2 bar (open symbols) (gradient membrane, 50 °C). For equivalent of β-Lg and time dependent data, see Supplementary Information.

It is well known that the transmembrane pressure has a major impact on the filtration performance [36]. Figure 4 shows the flux and the protein transmission as a function of the nominal pore size. The flux difference between Δp_TM_ = 1 bar and Δp_TM_ = 2 bar was below 10% for the pore sizes 0.14 µm, 0.2 µm, and 0.45 µm, which indicates that the limiting flux was already reached at 1 bar of the transmembrane pressure. Even though the applied transmembrane pressure did not have a great impact on the flux, the IgG transmission decreased to 50% when increasing the pressure from Δp_TM_ = 1 bar to Δp_TM_ = 2 bar, which is in agreement with previously reported data for the major whey proteins [16,37,38]. The decrease can be attributed to the higher compactness of the deposit layer and, therefore, a reduction of the porosity of the fouling layer takes place, which in turn results in a reduction of protein transmission.

**Figure 5:**
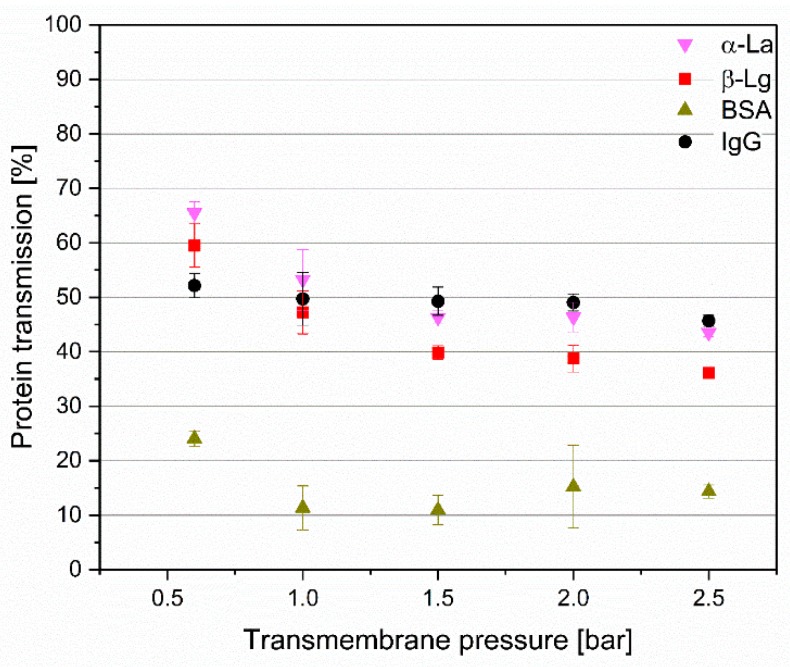
Protein transmission of IgG, α-La, β-Lg, and blood serum albumin (BSA) as a function of the transmembrane pressure at a pore size of 0.14 µm (gradient membrane, 50 °C). Error bars represent standard error of triplets.

The impact of the transmembrane pressure on IgG and β-Lg transmission was studied in more detail for the chosen pore size of 0.14 µm (Figure 5). For a better comparison, the protein transmission of the second largest whey protein fraction α-La as well as blood serum albumin (BSA) as a reference for a larger whey protein was studied. The transmission of BSA was around 10% to 25% depending on the applied pressure, which is in the range of previously reported data [36]. Even though the molecular mass (66.4 kDa) [39] and mean hydrodynamic diameter (6.4 nm) [23] of BSA are lower than IgG with (146–163 kDa) [3] and 10.4 nm [23], the transmission of IgG (45–55%) was significantly higher than that of BSA (10–20%). This substantiates the hypothesis formulated in chapter 3.1 that the size must be responsible for comparatively good transmission of IgG, which should be studied in more detail. For the other whey proteins, the transmission was similar and approximately constant with 40% for β-Lg and 45–50% for α-La at different transmembrane pressure levels (1.5 bar, 2.0 bar, and 2.5 bar). For the lower applied transmembrane pressure of 1.0 bar and 0.6 bar, the transmission was higher. This again can be attributed to the deposit layer, which is less intense and compact at the lower pressure due to the lower convective transport of particles to the membrane.

### 3.4. Impact of Temperature on IgG Transmission and Flux

**Figure 6:**
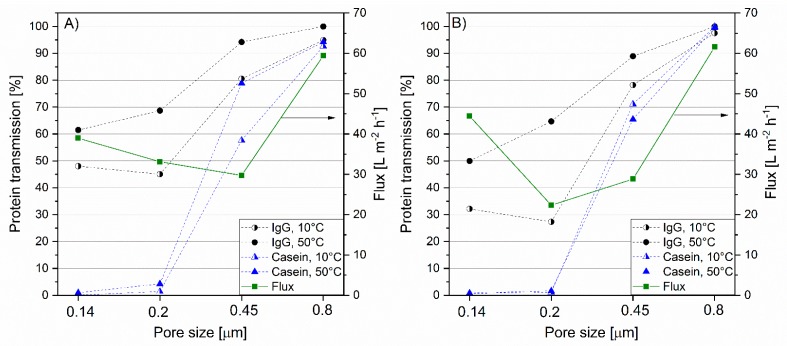
IgG and casein transmission plus flux as a function at 10 °C and 50 °C at Δp_TM_ = 1 bar (**A**) and Δp_TM_ = 2 bar (**B**) as a function of pore size at steady state conditions using gradient membranes. Arrows indicate the belonging of the flux curve to the right y-axis. For equivalent β-Lg and time-dependent data, see the Supplementary Information.

For microbiological reasons, milk protein fractionation with microfiltration is carried out at either cold temperatures around 10 °C [37,40], i.e., below the growth optimum of microorganism in milk or at 50–55 °C [12,37,41], i.e., above the microbiological growth optimum. Especially for ceramic membranes, higher temperatures are preferred due to the lower viscosity of the retentate and the inherent advantages during long production runs. Figure 6 shows the transmission of IgG and casein at 10 °C and 50 °C at Δp_TM_ = 1 bar (A), respectively. Δp_TM_ = 2 bar (B) as a function of the pore size. The casein transmission was similar at both temperatures. However, it should be noted that, during extended filtration times whereupon the permeate is collected and concentrated with an ultrafiltration unit, there might be an accumulation of β-casein at 10 °C in the whey [42,43]. The IgG transmission was 20% to 30% lower at 10 °C compared to 50 °C at the same operating conditions. A reason for the lower transmission could be due to the lower flow velocity in the laminar boundary layer of the membrane where fouling takes place. The thickness of this boundary can be estimated according to Equation (3) [44].

(3) $$\;\delta_{lam}=5\cdot \nu \cdot \sqrt{\rho /\tau_{w}}$$

At 10 °C, the kinematic viscosity of skim milk is about two times higher (1.48 × 10^−6^ m^2^ s^−2^ versus 7.84 × 10^−7^ m^2^ s^−2^ at 50 °C), which means the laminar boundary layer is approximately two times thicker (1.02 × 10^−5^ m versus 1.95 × 10^−5^ m). This, in turn, leads to a less turbulent flow profile directly above the membrane, which again effects the deposit layer and protein transmission [45]. The flux was at 39 L m^−1^ h^−1^ (Δp_TM_ = 1 bar), respectively, and 44 L m^−1^ h^−1^ (Δp_TM_ = 2 bar) for the pore size of 0.14 µm. Therefore, 35% or 43%, respectively, of the flux at 50 °C can be attributed to the higher viscosity and density at the lower temperature [37,45]. In addition, the same effect of the decreasing flux with increasing pore size from 0.14 µm to 0.2 µm and 0.45 µm was observed at 10 °C. In summary, the operating temperature of 10 °C is feasible for the fractionation of IgG and casein using ceramic membranes. Even though the low temperature will extend the filtration time, respectively, in the required filtration area, it could be necessary to use a lower temperature than 50 °C over extended filtration times since bovine antibodies are known to be heat sensitive [8].

## 4. Conclusions

The aim of this work was to determine whether it is possible to use ceramic microfiltration for the fractionation of casein micelles and bovine IgG and, if so, which pore size and operating conditions should be used. Membranes with pore sizes of 0.2 µm, 0.45 µm, and 0.8 µm, irrespective of membrane type (standard or gradient) and operating parameters such as transmembrane pressure (1 bar or 2 bar) and temperature (10 °C or 50 °C), were inapplicable for the fractionation of IgG and casein due to casein transmission. In contrast, it was found that ceramic gradient membranes with a pore sizes of 0.14 µm featured IgG-transmission rates between 45% to 62% depending on the process conditions while reducing the casein fraction below 1% in the permeates. The transmission of IgG taken as a model protein for immunological active proteins in whey was observed to be similar than that of the much smaller globular major whey protein fractions β-Lg and α-La while the intermediate-sized and ellipsoidal BSA showed a lower transmission. Transferring the observed results to a continuous process offers new possibilities for production of immunoglobulin enriched supplements using industrial filtration equipment on a large scale.

## Supplementary Materials

The following are available online at http://www.mdpi.com/2304-8158/7/7/101/s1.
