# Supplementary material for: Concentration of Immunoglobulins in Microfiltration Permeates of Skim Milk: Impact of Transmembrane Pressure and Temperature on the IgG Transmission Using Different Ceramic Membrane Types and Pore Sizes
Source: Foods. 2018 Jun 28;7(7):101. doi: 10.3390/foods7070101 (PMC6068916; doi:10.3390/foods7070101)
Supplement: Supplementary file 1 [file foods-07-00101-s001.pdf]

Supplementary Material: Concentration of Immunoglobulins in Microfiltration Permeates of Skim Milk: Impact of Transmembrane Pressure and Temperature on the IgG Transmission Using Different Ceramic Membrane Types and Pore Sizes

Hans-Jürgen Heidebrecht [1,*], José Toro-Sierra [1,2], Ulrich Kulozik [1,3]

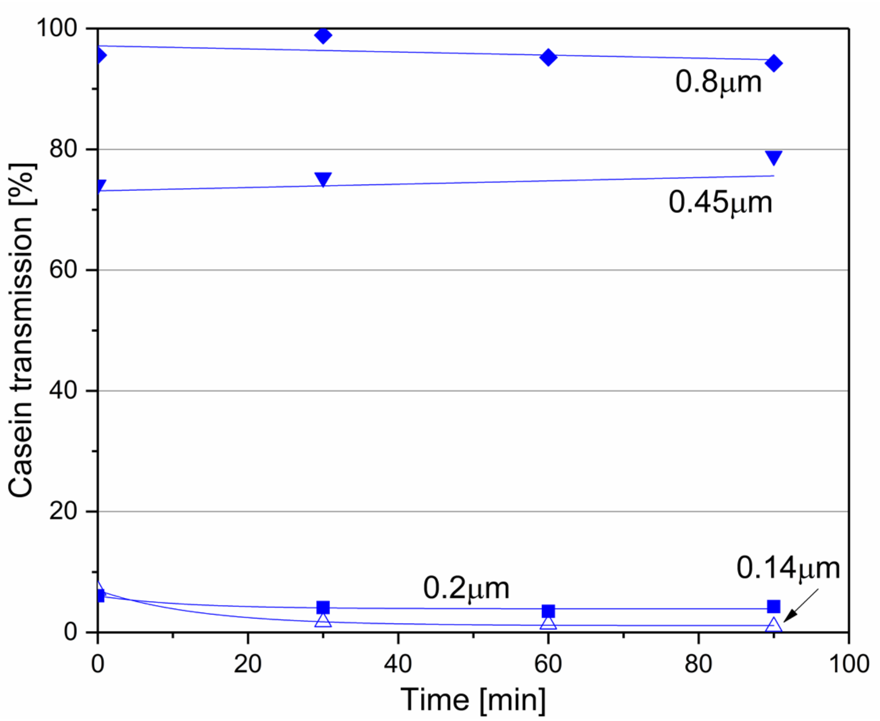

Figure S1. Casein transmission as function of time at 50 °C, 1 bar TMP, using ceramic gradient membranes.

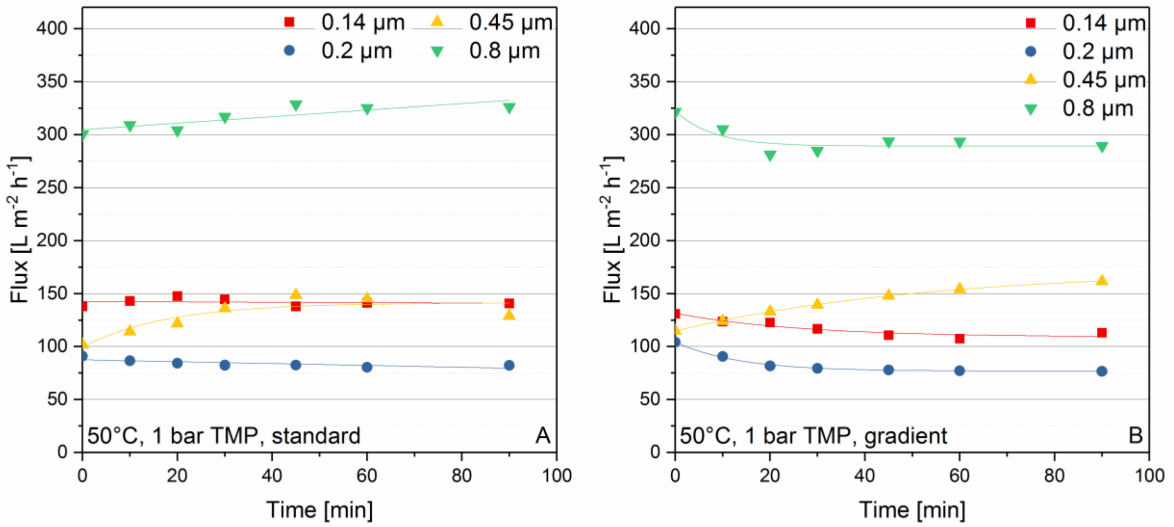

Figure S2. Comparison of flux as function of time at 50 °C, 1 bar Δp_{TM} using standard membranes (A) and gradient membranes (B).

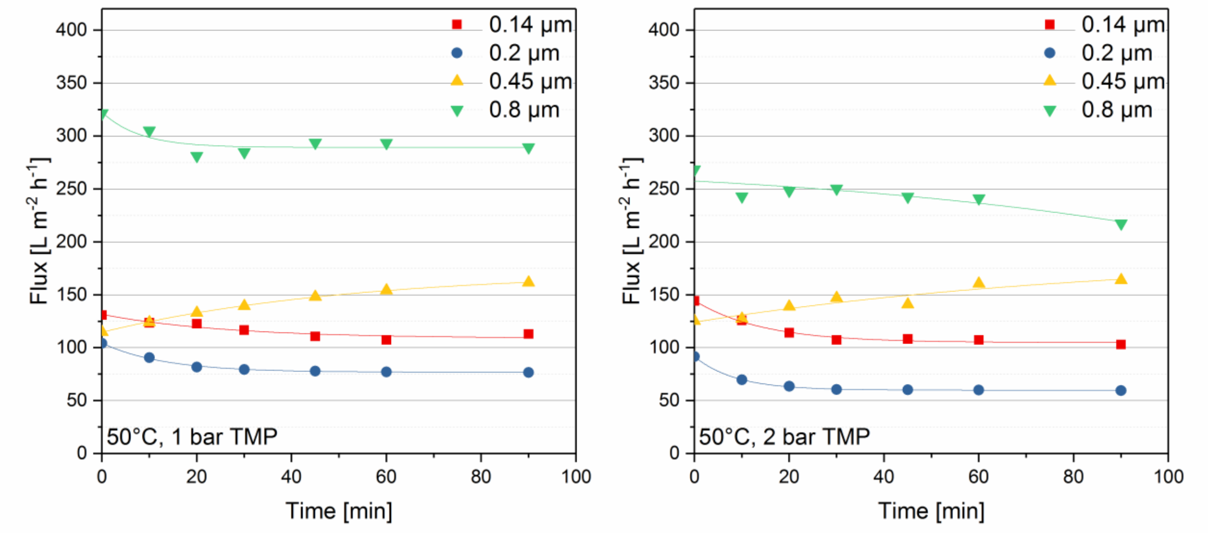

Figure S3. Comparison of flux as function of time at 50 °C, using gradient membranes at Δp_{TM} = 1 bar (A) and Δp_{TM} = 2 bar (B).

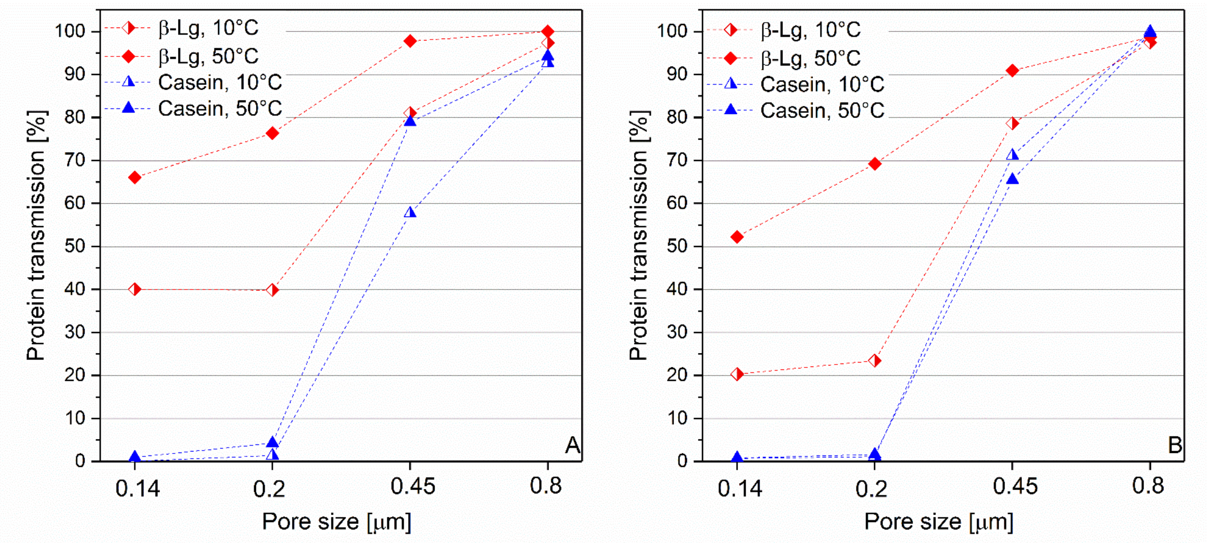

Figure S4. Comparison of β-Lg and casein transmission at 10 °C and 50 °C at Δp_{TM} = 1 bar (A) and Δp_{TM} = 2 bar (B) as function of pore size at steady state conditions.

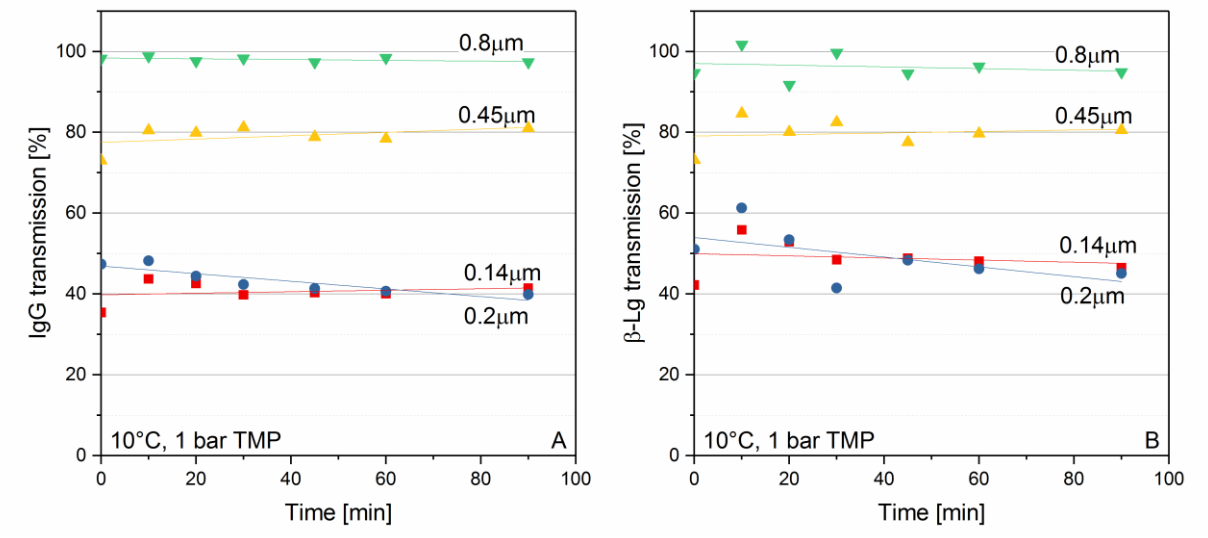

Figure S5. Comparison of IgG (A) and β-Lg (B) transmission as function of time at 10 °C, using gradient membranes at Δp_{TM} = 1 bar

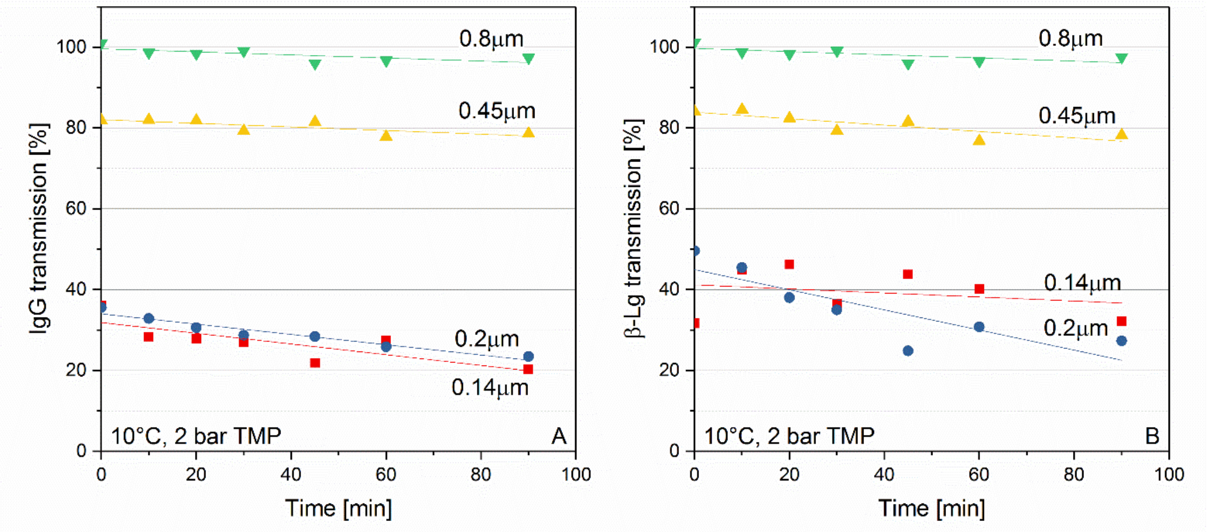

Figure S6. Comparison of IgG (A) and β-Lg (B) transmission as function of time at 10 °C, using gradient membranes at Δp_{TM} = 2 bar.

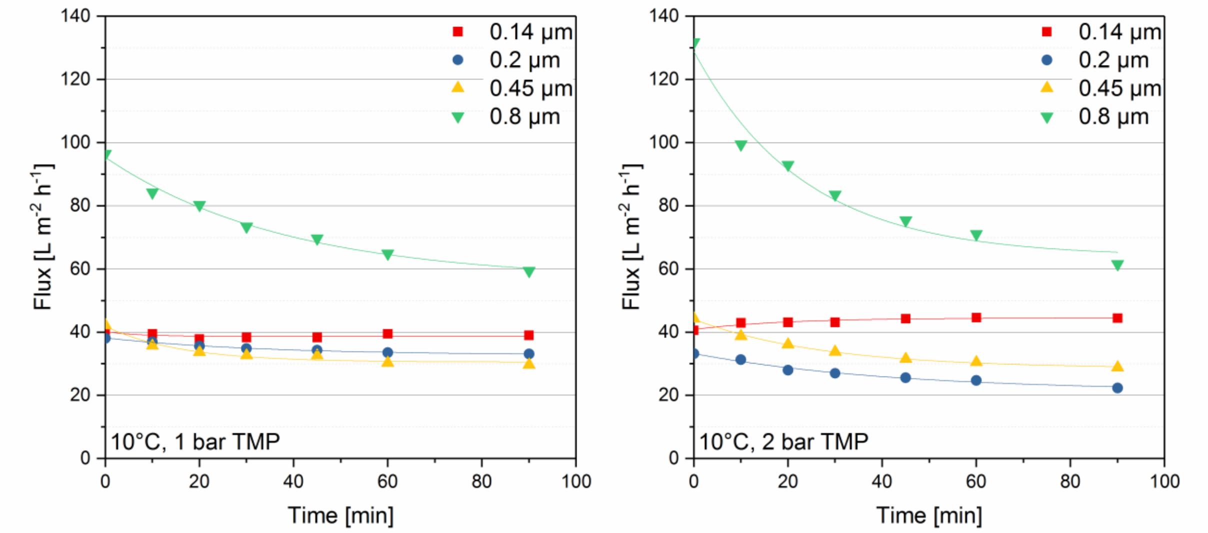

Figure S7. Comparison of flux as function of time at 10 °C, Δp_{TM} = 1 bar (A) and Δp_{TM} = 2 bar (B) using gradient membranes .
